# Supplementary material for: Influence of Population Size, the Human Development Index and the Gross Domestic Product on Mortality by COVID-19 in the Southeast Region of Brazil
Source: Int J Environ Res Public Health. 2022 Nov 4;19(21):14459. doi: 10.3390/ijerph192114459 (PMC9658565; doi:10.3390/ijerph192114459)
Supplement: Supplementary file 1 [file ijerph-19-14459-s001.zip › ijerph-1896175-supplementary-Table S1.pdf]

Table S1. Absolute proportion of municipalities of the four states of the Brazilian Southeast region, according to population size (POP); gross domestic product (GDP); and Municipal Human Development Index (HDI-M), over the course of 62 weeks of the pandemic.

		Proportion of municipalities of the Brazilian Southeast region				
		**Espírito Santo (n=78)**	**Minas Gerais (n=853)**	**Rio de Janeiro (n=92)**	**São Paulo (n=645)**	**Total (n=1668)**
	**Total of inhabitants**	**4.018.650**	**21.168.791**	**17.264.943**	**45.919.049**	**88.371.433**
**Population**	**Small**	66 (84.6%)	782 (91.7%)	54 (58.7%)	506 (78.5%)	1408 (84.4%)
	**Medium**	2 (2.56%)	38 (4.5%)	8 (8.7%)	58 (9.0%)	106 (6.4%)
	**Big**	9 (11.54%)	29 (3.4%)	23 (25.0%)	72 (11.2%)	133 (8.0%)
	**Metropolis 0,5 - 1 million**	1 (1.28%)	3 (0.35%)	5 (5.43%)	6 (0.93%)	15 (0.9%)
	**Metropolis 1 - 2 million**	-	-	1 (1.09%)	2 (0.31%)	3 (0.18%)
	**Metropolis > 2 million**	-	1 (0.12%)	1 (1.09%)	1 (0.16%)	3 (0.18%)
**Number of Inhabitants (×100.000)**	**Small**	12.7 (31.6%)	86.6 (40.9%)	12.3 (7.1%)	70 (15.2%)	181.7 (20.6%)
	**Medium**	1.3 (3.2%)	27.5 (13%)	5.6 (3.2%)	40.2 (8.8%)	74.6 (8.4%)
	**Big**	21 (52.3%)	53.2 (25.1%)	43.9 (25.4%)	157.1 (34.2%)	275.2 (31.1%)
	**Metropolis 0,5 - 1 million**	5.2 (12.9%)	19.2 (9.1%)	32.7 (18.9%)	43.6 (9.5%)	100.8 (11.4%)
	**Metropolis 1 - 2 million**	-	-	10.8 (6.3%)	25.8 (5.6%)	36.7 (4.2%)
	**Metropolis > 2 million**	-	25.1 (11.9%)	67.2 (38.9%)	122.5 (26.7%)	214.8 (24.3%)
**GDP**	**Very low**	-	176 (20.6%)	-	6 (0.93%)	182 (10.91%)
	**Low**	67 (85.9%)	563 (66%)	61 (66.3%)	396 (61.4%)	1087 (65.2%)
	**Middle**	7 (8.97%)	90 (10.6%)	17 (18.5%)	194 (30.1%)	308 (18.5%)
	**High**	2 (2.56%)	15 (1.76%)	9 (9.78%)	31 (4.81%)	57 (3.42%)
	**Very high**	2 (2.56%)	9 (1.06%)	5 (5.43%)	18 (2.79%)	34 (2.04%)
**Number of inhabitants/GDP**	**Very low**	-	16.9 (8%)	-	2.3 (0.5%)	19.2 (2.2%)
	**Low**	26.1 (64.9%)	108.8 (51.4%)	60.3 (34.9%)	99.8 (21.7%)	295.1 (33.4%)
	**Middle**	9.6 (23.9%)	80.7 (38.1%)	92.7 (53.7%)	312.2 (68%)	495.2 (56%)
	**High**	4 (10%)	3.1 (1.5%)	16.9 (9.8%)	23.9 (5.2%)	47.9 (5.4%)
	**Very high**	0.46 (1.1%)	2.2 (1%)	2.7 (1.6%)	21 (4.6%)	26.4 (3%)
**IDH-M**	**Low**	-	73 (8.56%)	-	-	73 (4.38%)
	**Medium**	46 (58.97%)	548 (64.24%)	35 (38.04%)	66 (10.23%)	695 (41.67%)
	**High**	30 (38.46%)	230 (26.96%)	55 (59.78%)	556 (86.2%)	871 (52.22%)
	**Very high**	2 (2.56%)	2 (0.23%)	2 (2.17%)	23 (3.57%)	29 (1.74%)
**Number of inhabitants/IDH-M**	**Low**	-	6.3 (3%)	-	-	6.3 (0.7%)
	**Medium**	9.1 (22.6%)	60 (28.3%)	18 (10.4%)	8.4 (1.8%)	95.5 (10.8%)
	**High**	22.5 (56%)	119.4 (56.4%)	149.4 (86.5%)	252.7 (55%)	544 (61.6%)
	**Very high**	8.6 (21.4%)	26.1 (12.3%)	5.3 (3.1%)	198.1 (43.1%)	238 (26.9%)

Legend: POP classification: Small cities (under 50,000 inhabitants); medium cities (50,000 to 100,000 inhabitants); big cities (100,000 to 500,000 inhabitants); and metropolises (over 500,000 inhabitants). GDP classification: Very low GDP (lower than BRL10,000); low GDP (BRL10,001 to BRL30,000); middle GDP (BRL30,001 to 60,000); high GDP (BRL60,001 to 100,000); and very-high GDP (higher than BRL100,000). HDI-M classification: very low HDI-M (0 to 0.499); low HDI-M (0.500 to 0.599); medium HDI-M (0.600 to 0.699); high HDI-M (0.700 to 0.799); and very high HDI-M (0.800 to 1.0).
