# Supplementary figures and images for: Influence of Population Size, the Human Development Index and the Gross Domestic Product on Mortality by COVID-19 in the Southeast Region of Brazil
Source: Int J Environ Res Public Health. 2022 Nov 4;19(21):14459. doi: 10.3390/ijerph192114459 (PMC9658565; doi:10.3390/ijerph192114459)

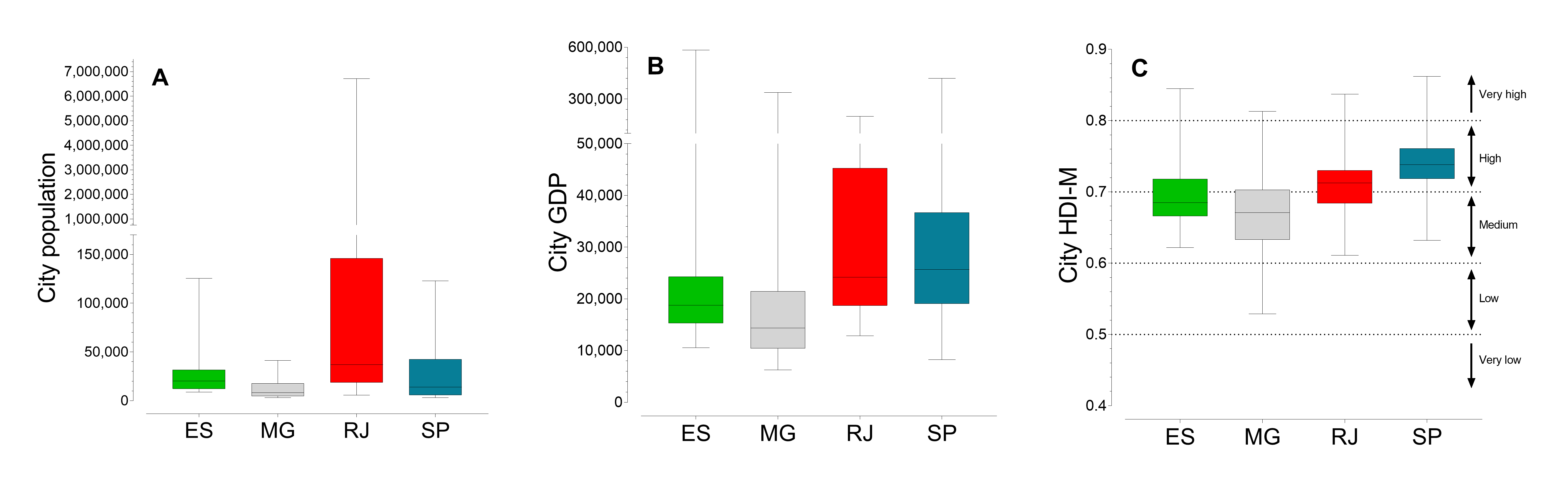

Supplement: Supplementary file 1 [file ijerph-19-14459-s001.zip › ijerph-1896175-supplementary-Figure S1.tif]
